# Activation of the Nrf2 Pathway as a Therapeutic Strategy for ALS Treatment

**DOI:** 10.3390/molecules27051471

**Published:** 2022-02-22

**Authors:** Liaisan Arslanbaeva, Marco Bisaglia

**Affiliations:** 1 Department of Biology, University of Padua, 35131 Padua, Italy; 2 Center Study for Neurodegeneration (CESNE), University of Padua, 35131 Padua, Italy

**Keywords:** amyotrophic lateral sclerosis, astrocytes, motoneurons, non-cell-autonomous toxicity Nrf2, Nrf2-activating therapy

## Abstract

Amyotrophic lateral sclerosis is a progressive and fatal disease that causes motoneurons degeneration and functional impairment of voluntary muscles, with limited and poorly efficient therapies. Alterations in the Nrf2-ARE pathway are associated with ALS pathology and result in aberrant oxidative stress, making the stimulation of the Nrf2-mediated antioxidant response a promising therapeutic strategy in ALS to reduce oxidative stress. In this review, we first introduce the involvement of the Nrf2 pathway in the pathogenesis of ALS and the role played by astrocytes in modulating such a protective pathway. We then describe the currently developed activators of Nrf2, used in both preclinical animal models and clinical studies, taking into consideration their potentialities as well as the possible limitations associated with their use.

## 1. Introduction

Amyotrophic lateral sclerosis (ALS) is an adult-onset disease characterized by the progressive degeneration of motoneurons (MNs) in the brain and spinal cord, which leads to a rapid and progressive paralysis of skeletal muscles and, ultimately, to death due to respiratory failure, within 3 to 5 years after the onset of disease symptoms [1,2]. ALS has an incidence of about 1–2 and a prevalence of approximately 5 per 100,000 individuals [2]. Most ALS cases are sporadic, but about 5–10% of ALS cases have a genetic origin and are generally inherited in an autosomal dominant way with high penetrance [2,3,4]. No effective cure is currently available for ALS patients. The therapeutic strategies are mainly based on the use of riluzole or edaravone, with very limited efficacy, and offer only minimal palliative care.

ALS is a complex and still elusive disorder in which both genetic susceptibilities and environmental factors seem to contribute to disease onset. Mitochondrial dysfunction, oxidative stress (OS), impaired RNA regulation, protein misfolding, altered autophagy, glutamate excitotoxicity, and neuroinflammation are all factors involved in the pathogenesis of ALS, even though it is unclear whether they play a role in all patients [2]. Although ALS is characterized by the selective loss of MNs, accumulating data support a central role of non-cell-autonomous processes, resulting from glia dysfunction, in MN degeneration [5,6]. Considering the multiple pathophysiological pathways associated with the disease and the pathological role exerted by glial cells, the activation of the nuclear factor erythroid 2-related factor 2 (Nrf2) pathway appears a very promising therapeutic strategy to cope with ALS, that deserves high consideration.

Nrf2 is a master regulator of multiple cytoprotective responses, which participates in the transcription of hundreds of genes involved in the regulation of glycolysis, pentose phosphate pathway, fatty acid, glutamine, and glutathione metabolism, as well as in iron and redox homeostasis. Nrf2 is found almost ubiquitously in all cell types even though, within the brain, its transcript is found at higher levels in astrocytes and microglia than neurons [7]. The intracellular protein levels are maintained at a low basal level in unstressed cells due to its well-established proteasomal degradation, which is mediated by Kelch-like ECH-associated protein 1 (Keap1) [8,9], whose transcript is also more expressed in astrocytes and microglia than neurons [7].

In the present review, we will analyze the vulnerability of MNs to OS conditions and the protective mechanisms against reactive oxygen species (ROS) mediated by glia and Nrf2 activation. We will also discuss the possible therapeutic use of small-molecule activators of the Nrf2 pathway.

## 2. Motoneurons Vulnerability to Oxidative Stress

Nowadays, it is well accepted that aging is associated with increasing levels of OS [10] enhancing in such a way the risk of many diseases, including ALS, where impaired ROS imbalance is a part of pathomechanism [8,11]. OS causes covalent modifications in proteins, lipids, and DNA [12]. MNs are large cells with axon extensions that reach far distant locations from the motor cortex and spinal cord to target muscles. Due to high metabolic rate and mitochondrial respiration, low levels of catalase and GSH, high activity of NADPH oxidases and decreased Nrf2 protein levels, MNs are particularly subjected to OS [13,14,15,16], as summarized in Table 1.

Accordingly, aberrant oxidative damage, including signs of lipid peroxidation, has been documented in biosamples and post-mortem tissues from patients with either sporadic or familial forms of ALS [18], as well as in both cellular and animal models of ALS [11,19,27,28]. In addition, the GSSG/GSH ratio, another indicator of OS, has been reported to be increased in ALS mouse models [29] and ALS patients [18,20]. Interestingly, lipid peroxidation has been shown to promote superoxide dismutase 1 (SOD1) aggregation, which is involved in some familial forms of ALS and could contribute to the pathology [30].

Different dysregulated ROS-generating sources can result in the generation of OS in MNs, which include mitochondrial dysfunction and defective respiration [8,11], hyperactivation of glia with neuroinflammation [4,31], and impaired Nrf2 pathway [13,14]. Moreover, as a result of lipid peroxidation and iron accumulation, ferroptosis has been reported to induce MN degeneration. [32,33,34]. In fact, accumulation of iron was observed in neurons of ALS mouse models [35,36], as well as in the spinal cord and different brain regions of ALS patients [36,37]. Dysregulation of iron-binding proteins was also described in ALS mouse models [17]. Moreover, depletion of glutathione peroxidase 4, an enzyme involved in the cellular protection against ferroptosis, was found in post-mortem samples from ALS patients and described as an early feature of mouse ALS models [17]. Despite the clear and well-accepted role exerted by OS in ALS, it is, however, unclear whether OS is a primary or a secondary event in the pathogenesis of ALS. 

## 3. Antioxidant Therapy in ALS

Antioxidant medications are widely accessible, are usually well-tolerated without serious side effects, and very often are consumed without medical prescription. Considering the aforementioned indication of the role of OS in ALS pathogenesis, the use of antioxidants to cope with the disease has been largely explored with results that, unfortunately, are rather disappointing. A first promising indication on the protective role of antioxidants came from the observation that a diet rich in natural antioxidants decreases the incidence rate of age-related disorders [38,39], even though it does not seem to be effective in rapidly progressing diseases [40]. Moreover, an analysis of the dietary regimen at the time of ALS diagnosis showed that healthy nutrients and antioxidants are in general associated with better patient respiratory and physical functions and lower level of disability around the time of diagnosis [41]. In contrast with these indications, other studies, however, showed that antioxidants are not effective in reducing ALS symptoms and/or extending the life of ALS patients [42]. In fact, despite the slowness in disease progression that was observed in animal models of ALS following the treatment with antioxidants, such as coenzyme Q10 and vitamin E, subsequent clinical trials did not show any significant therapeutic effects of these antioxidants in human patients [43]. The treatment with N-acetylcysteine and GSH also showed no protective effect on ALS patients [44,45]. Moreover, a study focused on the endogenous levels of vitamins A, C, and E in the sera of ALS patients and control individuals reported no changes between these 2 groups, indicating that endogenous blood antioxidants do not participate in preventing ALS onset and slowing down the disease progression [18]. The only exception seems to be represented by edaravone, a ROS scavenger in the central nervous system, which, however, possesses a very limited efficacy against ALS [46,47]. All these data support the idea that antioxidants from supplements or food are poorly effective in reducing OS in ALS patients.

## 4. Nrf2 Involvement in ALS

An alternative therapeutic approach in ALS could be represented by protein targeting, and, in this frame, the Nrf2 pathway appears very promising since it exerts an important cytoprotective role by regulating the transcription of more than 500 genes [48]. Not only has Nrf2 expression been described to decline with aging [49], but the Nrf2 pathway seems to be impaired in ALS patients as well as in cell cultures and animal models of the disease. Accordingly, post-mortem analyses showed decreased Nrf2 protein levels in the motor cortex and spinal cord of ALS patients compared to controls, in contrast to Keap1 mRNA that was found elevated in the motor cortex [50]. Moreover, iPSC lines derived from skin fibroblasts of C9orf72 mutated ALS patients were demonstrated to undergo age-dependent OS, due, in part, to reduced levels of glutathione synthetase and peroxiredoxins, whose expression is under the control of the Nrf2-pathway [51]. Nrf2 protein levels were also shown to be reduced in primary MN cultures from SOD1^G93A^ transgenic mice compared to wild-type animals [52], while the overexpression of Nrf2 in NSC-34 SOD1^G93A^ cells was able to significantly decrease OS and increase cell survival [53]. 

In another study based on the use of fibroblasts carrying the TDP-43^M337V^ mutation and NSC-34 cells carrying the TDP-43^Q331K^ mutation, the investigators described an alteration in the Nrf2-mediated response, that was associated with a dysregulation in Nrf2 mRNA metabolism. More specifically, the expression of TDP-43 mutants was found to alter the expression and localization of heterogeneous nuclear ribonucleoprotein K, which is normally localized to the nucleus where it participates in mRNA splicing, mRNA stability, regulation of transcription, and translation. The binding of ribonucleoprotein K with Nrf2 transcript was then associated with an impaired translation of Nrf2 mRNA, leading to an insufficient antioxidant response and motoneuron degeneration [27].

Interestingly, a correlation between Nrf2 expression levels in MNs isolated from SOD1 transgenic mice with distinct genetic backgrounds (C57 and 129Sv) and a different rate of disease progression was shown using transcriptome analysis. While MNs from the rapid ALS progressing 129Sv-SOD1^G93A^ strain expressed low Nrf2 level, elevated mRNA and protein expression levels of Nrf2 observed in C57-SOD1^G93A^ MNs at the onset of the disease appeared to be important in slowing disease progression [54]. Nonetheless, in contrast to these results, in other works performed using in vivo ALS mouse models, a direct role of Nrf2 in ALS pathogenesis was excluded. In fact, deletion of Nrf2 in different SOD1 mouse models (SOD1^G93A^, SOD1^G85R^ or SOD1^H46R^) has been shown not to drastically change the progression of the disease [55,56,57], and the overexpression of Nrf2 in MNs of SOD1^G93A^ mouse shifted the disease onset later but did not alter the survival [56].

Another aspect that should be considered is that the antioxidant response mediated by Nrf2 could be relevant only in specific forms of ALS, as suggested by analyses carried out on lymphoblasts from ALS patients [47]. Lymphoblasts are often used as ALS models since they recapitulate features of affected MNs, such as, for instance, alterations in TDP-43 homeostasis. The status of the Nrf2-ARE system was assessed in sporadic ALS and a SOD1-related familial form of the disease. The authors of the study found that Nrf2 mRNA levels were similar in both types of cells. However, increased levels of ROS in lymphoblasts from sporadic ALS subjects were able to activate the Nrf2 response by stimulating the expression of downstream proteins, while Nrf2 and NQO1 protein levels were lower in lymphoblasts derived from SOD1-related ALS subjects, suggesting that the Nrf2-mediated antioxidant response was activated in the sporadic ALS model, but not in the model of familial ALS. As a consequence, the authors concluded that the pharmacological modulation of Nrf2 for ALS should be personalized.

In conclusion, even though some aspects still need to be clarified, the activation of the Nrf2-mediated antioxidant response may serve as a therapeutic approach in ALS.

## 5. Role of Glial Cells in ALS

**Figure 1:**
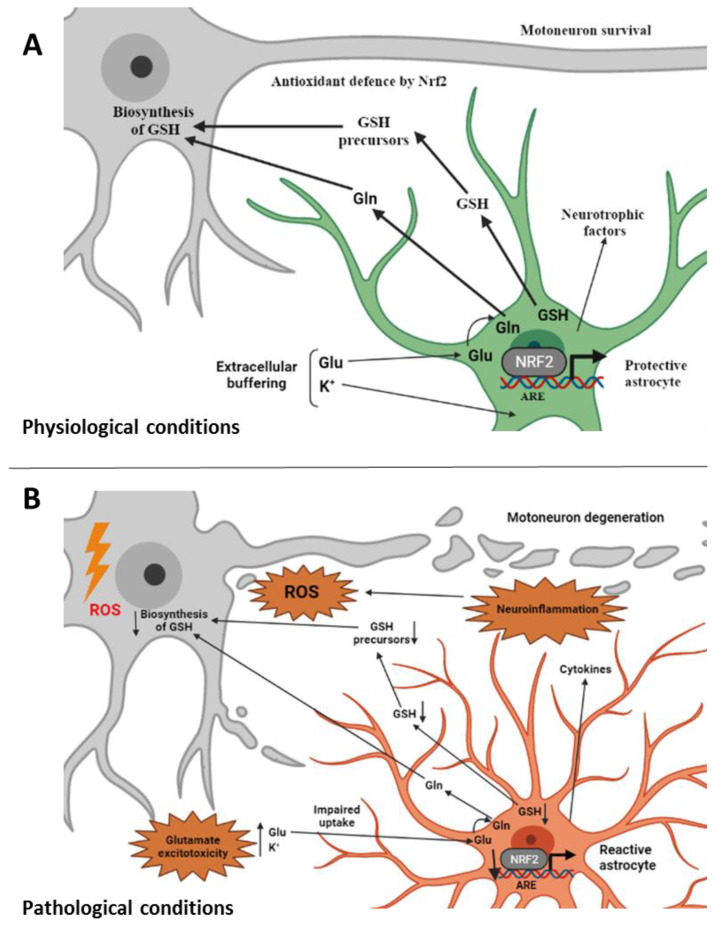
Involvement of non-cell-autonomous processes in ALS. (**A**) Under physiological conditions astrocytes perform a variety of neuroprotective tasks toward motoneurons from axon guidance and synaptic support, to the control of the blood brain barrier and blood flow, from the maintenance of extracellular potassium levels to the removal of the neurotransmitter glutamate. Astrocytes provide Nrf2-mediated antioxidant protection of motoneurons by supplying GSH and GSH precursors to motoneurons thus protecting them from oxidative stress [81]. (**B**) Under ALS-related pathological conditions, astrocytes change their morphology and properties and become “reactive astrocytes”. They release toxic factors and inflammatory mediators and promote glutamate excitotoxicity [16]. Impairment in the Nrf2 pathway at the astrocytic level, which does not allow the supply of GSH and its metabolites to motoneurons, is also associated with reactive astrocytes [1]. ALS, amyotrophic lateral sclerosis; ROS, reactive oxygen species; GSH, reduced glutathione; Gln, glutamine; Glu, glutamate; Nrf2, nuclear factor erythroid 2–related factor 2; ARE, antioxidant responsive element (created with BioRender.com, accessed on 17 February 2022).

Due to the specific degeneration of MNs that characterizes ALS, research mainly focused on the molecular pathways involved in the pathogenesis of the disease at the level of this specific neuronal population. However, in recent years, the role of non-cell-autonomous pathogenic mechanisms has become increasingly evident, highlighting the role of glial cells, and astrocytes in particular, in ALS pathology [16,51,58]. In astrocytes, a Nrf2-mediated response drives non-cell-autonomous protection of nearby neurons and ameliorates OS [14,59,60,61,62]. As represented in Figure 1, astrocytes are the major source of GSH in the brain through the activated Nrf2 pathway [63], and the transport of GSH precursors from astrocytes to MNs appears to be critical for the neuroprotective function of astrocytes [64,65]. Moreover, astrocytes were shown to protect MNs from OS through the Nrf2-mediated response [66]. The investigators evaluated both temporal and spatial changes of protein levels of Nrf2, Keap1, and other Nrf2-downstream genes such as heme oxygenase-1, thioredoxin, and heat shock protein 70 during MN degeneration in the spinal cord of an ALS mouse model. They found that the Keap1-Nrf2-ARE pathway did not induce the expression of target genes in spinal MNs, while the protective pathway was activated in glial cells at a late stage [66]. In the same line, while the total Nrf2 protein levels were found lower in ALS tissues (see the previous paragraph), higher astrocytic Nrf2 levels were detected compared to other cells [50]. Moreover, in the SOD1^G93A^-based ALS rat model, Nrf2 was found to localize in astrocytes where its levels were higher at the onset of the disease in comparison to the basal level of controls [67,68].

While the neuroprotective action of astrocytes is fundamental to preserve MN functionality, under certain conditions, astrocytes can become reactive supporting a neuroinflammatory response that could further promote neurodegeneration [65]. Interestingly, astrocytes have been described to become reactive also after brain injury [69], which has been indicated as a predisposing factor for ALS and could serve as a link to induce ALS [70]. The contribution of reactive astrocytes to ALS pathogenesis has been experimentally supported using in vitro cellular co-cultures. [11,61,69]. Moreover, chemical activation or overexpression of Nrf2 in astrocytes was shown to increase neuronal survival both in in vitro co-culture studies [68] and in an ALS mouse model [59].

While the involvement of astrocytes in the non-cell-authonomous pathogenic mechanisms associated with ALS has been the most investigated, other glial cells can participate in MN degeneration as well. For instance, the aberrant activation of microglia with subsequent ROS generation and neurotoxic factors secretion is a common hallmark of several neurodegenerative disorders, including ALS [5,71]. As aforementioned, within the brain, Nrf2 transcript is found at higher levels in astrocytes and microglia than neurons [72], so that the activation of the Nrf2-associated pathway could represent a potential therapeutic strategy to inhibit the microglia-dependent inflammatory response [31,71].

Altough their role in the disease is still under investigation and many mechanistic aspects need to be clarified, as recently reviewed, dysfunctional and degenerating oligodendroglial cells and loss of terminal schwann cells seem to contribute to the ALS pathology, representing additional potential therapeutic targets for designing effective therapies [73,74,75]. Interestingly, the overexpression of Nrf2 in Schwann cells through targeted muscular injections or the activation of the Nrf2 signaling pathway by the oral administration diphenyl diselenide have been described to mediate sciatic nerve recovery in rat models of diabetic peripheral neuropathy [76,77]. Moreover, several natural compounds have been shown to exert antioxidant effects in different immortalized schwann cells by activating the Nrf2 pathway [78,79,80].

From what has just been discussed it follows that, when defining a novel therapeutic strategy, it appears evident that not only the pharmacological target is important, but also the precise type of cells associated with that specific pharmacological target. For this reason, an Nrf2-mediated therapeutic approach for ALS treatment should also consider the involvement of non-cell-autonomous mechanisms in the disease [56]. Moreover, the nature of astrocytes and microglia might change during ALS progression and, in particular, before and after the onset of the pathological symptoms. Accordingly, during pre-symptomatic stages, astrocytes could support the survival of MNs till a metabolic threshold, which, once exceeded, triggers the onset of the disease. Microglia as well could be protective in an early stage of the disease while contributing to enhance MN death at the end-stage. It follows that an Nrf2-targeted approach is complicated because it depends on many variables. To be more effective, it should probably focus to activate protective astrocytes while reducing the levels of reactive ones. The activation of the Nrf2 pathway in protective astrocytes could then be able to counteract OS in MNs.

## 6. Nrf2-Targeted Pharmacological Approach

**Figure 2:**
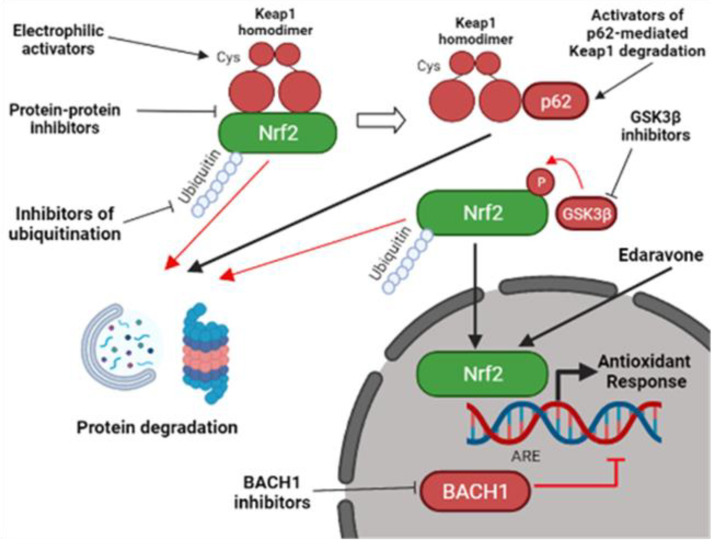
Pharmacological strategies to activate the Nrf2 pathway. Nrf2-activators do not usually target Nrf2 directly, but they function through the inhibition of different Nrf2-interactors. They can act by inhibiting Keap1-mediated Nrf2 degradation through the electrophilic modification of Keap1 cysteine residues or by interfering with the Nrf2-Keap1 protein-protein interaction. Other Nrf2-activators can promote Keap1 degradation by activating p62-mediated autophagy. Alternatively, Keap1-independent inhibitors can mediate Nrf2 activation. They include inhibitors of BACH1, an Nrf2-competitor for the ARE elements, inhibitors of GSK3β, a kinase involved in Nrf2 degradation, and inhibitors of other proteins involved in Nrf2 ubiquitination [1,86]. Edaravone, a FDA-approved ALS compound, was also shown to activate Nrf2 [87], but the mechanism is still elusive. Nrf2, nuclear factor erythroid 2–related factor 2; Keap1, Kelch-like ECH-associated protein 1; ARE, antioxidant responsive element; GSK3β, glycogen synthase kinase 3 beta; BACH1, BTB Domain And CNC Homolog 1 (created with BioRender.com, accessed on 27 January 2022).

Even with the aforementioned issues, the pharmacological activation of the Nrf2 pathway still appears as a promising therapeutic strategy to treat disorders where OS is involved [82]. Accordingly, the FDA-approved treatment for ALS patients based on edaravone has been shown in preclinical studies to increase Nrf2 protein levels [83,84]. As summarized in Figure 2, since the mechanism of Nrf2 post-transcriptional regulation is complex, the development of Nrf2 activators is mainly based on different approaches and strategies, which do not directly target Nrf2, but other proteins involved in the regulation of Nrf2 protein levels [85]. 

The first approach is based on the interaction with Keap1, the principal negative regulator of Nrf2, that targets the transcription factor for ubiquitination and proteasomal degradation [81]. Currently developed Keap1 inhibitors are divided into 2 types: electrophilic and protein–protein interaction (PPI) inhibitors [1]. The second approach is based on tight regulation of Nrf2 by other additional proteins which up-regulate or down-regulate Nrf2 levels [88]. 

## 7. Electrophilic Inhibitors of Keap1

Keap1 is a redox sensor, which contains 27 cysteine residues. Elevated ROS levels and covalent cysteine modifiers can alter Keap1 structure and promote Nrf2 activation [82]. Usually, electrophilic pharmacological Keap1 inhibitors covalently modify 3 cysteine residues, i.e., Cys-151, Cys-273, or Cys-288, most commonly Cys-151, promoting a conformational change of Keap1, which induces the dissociation of Nrf2 from the Nrf2-Keap1 complex [1,89,90]. Many natural compounds have been described to activate Nrf2 through electrophilic modification of Keap1 [85]. Food- and plant-derived components, such as sulforaphane, curcumin, or resveratrol, which activate Nrf2 in such a way, have been proposed to possess several advantages in comparison to synthetic compounds since they are already present in the human diet and they have been extensively used for the treatment of different diseases because of their antioxidant and anti-inflammatory actions with minimal side effects [91]. The antioxidant effects of natural compounds and natural compounds-derived molecules were mainly analyzed using both cellular and mouse models of ALS [92,93,94,95,96,97]. Some electrophilic Nrf2 activators also entered clinical trials with promising results, even though some of them, like sulforaphane and bardoxolone, were rejected due to the presence of side effects [90] and others, like dimethylfumarate (DMF), showed no improvement in the primary endpoint [98]. Nanocurcumin, a more soluble form of curcumin, has been tested either in multiple sclerosis patients or in sporadic cases of ALS in association with riluzole. In the former study, the molecule was demonstrated to reduce the level of pro-inflammatory cytokines, while in the latter, it was shown to be safe and improve the patients’ survival as an add-on treatment [43,99]. Future studies with larger sample sizes and longer duration are needed to confirm these findings. Clinical trials evaluating the effects of curcumin and resveratrol taken together or CC100 (caffeic acid) are currently ongoing [100,101]. The repurposing of the already FDA-approved Nrf2 activator, fingolimod phosphate, which is already used for the treatment of multiply sclerosis, and showed efficacy on ALS in vivo models, could serve as an additional therapeutic approach [81].

The list of electrophilic Keap1-inhibitors is continuously growing. However, most of them have not evolved beyond proof-of-concept studies because of the long time required to characterize their pharmacodynamics properties, clinical safety, and efficacy [82].

## 8. Protein–Protein Interaction (PPI) Inhibitors of the Keap1-Nrf2 Complex

In order to avoid off-target toxicity derived from electrophilic compounds, a number of groups developed a different approach based on non-electrophilic non-covalent compounds that competitively block the protein–protein interaction between Keap1 and Nrf2 [82]. Initially, drug discovery was mainly focused on the development of truncated Nrf2 peptides for docking of Keap1 [82,85]. However, peptide-based inhibitors possess low cellular permeability, poor stability, and low activity. As a consequence, other groups evaluated small interfering molecules with high affinity for Keap1 [102]. In addition to easily crossing the BBB, small-molecule drugs offer simpler drug design, predictable pharmacokinetics, and pharmacodynamics, higher stability, and bioavailability [103]. Amongst the natural compounds, a non-electrophilic compound called geopyxin F was isolated from the endolichenic fungal strain *Geopyxis* sp. and was shown to activate Nrf2 in cell lines [90]. Although PPI molecules possess improved selectivity compared to electrophilic ones, they also could have off-target effects. First, as Keap1 may interact with other proteins, PPI inhibitors with high affinity for Keap1 could induce the dissociation of other Keap1 partners. Alternatively, PPI inhibitors could bind with high affinity with Keap1 partners inhibiting their functions. In both cases, subsequent unknown biological effects could arise [72]. A second very relevant issue is that Keap1 binds Nrf2 through its C-terminal Kelch domain, and dozens of human proteins with Kelch domain structurally very similar to the one in Keap1 exist [86]. It is worth mentioning that, at present, no PPI inhibitor has entered clinical trials [90].

## 9. Other Ways for Nrf2 Activation

Several alternative ways can also be exploited to stimulate the Nrf2 pathway. Nrf2 activation can be achieved (i) by the inhibition of Nrf2 transcriptional competitors, such as the transcriptional factor BTB domain and CNC homolog 1 (BACH1), (ii) by the degradation of Keap1 mediated by the protein p62 or (iii) by the inhibition of proteins involved in Nrf2 degradation, such as glycogen synthase kinase 3 beta (GSK3β), β-Transducin Repeat Containing E3 Ubiquitin Protein Ligase (βTrCP), CR6-interacting factor 1(CRIF1), 3-hydroxy-3-ethylglutaryl-CoA reductase degradation protein 1 (HRD1), and WD repeat protein (WDR23) [1,82].

Transcriptional activity of Nrf2 could be elevated by inhibiting the protein BACH1, which competes with Nrf2 in binding ARE elements leading to the suppression of the Nrf2-mediated antioxidant response. For this reason, BACH1 may represent a promising target of drug development in order to activate the Nrf2 pathway [86]. Interestingly, several studies used synthetic small molecules to inhibit BACH1, both in vitro and in vivo, and some of them were tested in clinical trials to treat several disorders, even though none of them was directed against ALS [1].

The autophagy–adaptor protein p62 promotes Keap1 degradation and it could also be the object of a therapeutic approach [104]. Rapamycin and trehalose were shown to activate Nrf2 in a p62-mediated manner [104,105] and reached clinical trials for different disorders, such diabetes mellitus, systemic lupus erythematosus, and autosomal dominant polycystic kidney disease [82]. Interestingly, rapamycin has also been evaluated in a phase 2 clinical trial based on 63 ALS patients (NCT03359538), but results have not been published yet. It should be noticed that p62 up-regulation might not always be beneficial to treat ALS. In fact, from postmortem analyses, p62-positive aggregates were found to accumulate in the spinal cord and brain of ALS patients and the protein fraction found in the insoluble aggregates was shown to be unable to activate autophagy and the Nrf2-pathway [106]. Moreover, p62 overexpression in the SOD^H46R^ ALS mouse model was shown to accelerate the onset of the disease and shorten lifespan [107]. The application of inhibitors of GSK3β, which is involved in Nrf2 degradation, could represent an additional clinical strategy. GSK3β is a serine/threonine protein kinase, which has been reported to play essential roles in glycogen metabolism, cell proliferation, apoptosis, and other functions within the cell. GSK3β phosphorylates Nrf2 and promotes its ubiquitination and proteasomal degradation [85,86]. Activation of GSK3β has been described in a variety of neurodegenerative diseases, also using in vitro and in vivo ALS models and several GSK3β inhibitors have been tested in clinical trials related to Alzheimer’s disease and ALS [108]. Even though the contribution of GSK3β inhibition in the activation of the Nrf2 pathway needs to be better clarified, the use of GSK3β inhibitors as a co-targeting approach together with Keap1 inhibition has been recently suggested [86]. It should be noticed, however, that GSK3β inhibition could affect other signaling and metabolic pathways with subsequent adverse side effects. Other proteins involved in the Nrf2 ubiquitination and subsequent proteasomal degradation, such as HRD1, βTrCP, CRIF1 and WDR23, are currently under investigation for their potential to interfere with ALS progression [82,86]. It is worth mentioning here that the FDA-approved compound edaravone has been shown to increase expression of Nrf2 although the mechanism has not been defined yet [84,87,109]. Although the efficacy of this drug is very limited, its mechanism of action could be exploited to design more effective molecules.

Table 2 summarizes Nrf2 activators belonging to the different approaches described in the text.

## 10. Issues in Drug Discovery Related to ALS

The development of new drugs is a lengthy and costly process, taking years to complete the numerous steps required, starting from the definition of the therapeutic target to clinical trial and, eventually, the FDA approval. In addition, the treatment of ALS is complicated because the disease is heterogeneous. The still elusive pathophysiology of ALS makes target identification tough and most clinical trials directed against ALS have failed, with few of them that are still ongoing. There are challenging obstacles on the way of drug development against ALS related to the selection of the therapeutic target, model of preclinical studies, drug development, and translation of preclinical studies to humans. The activation of the Nrf2-mediated antioxidant response seems to be a promising approach to treat ALS. Accordingly, the Nrf2 pathway in human bronchial epithelial cells was demonstrated to be significantly down-regulated in adults older than 60 years, compared to the younger ones [118], and this seems related to the fact that the expression of BACH1, an Nrf2 competitor, increases with age suggesting that Nrf2 down-regulation derives from the BACH1-mediated suppression of ARE-genes. It is possible that a similar mechanism also happens at the neuronal level. Even with these premises, it is however possible that a strategy of Nrf2 activation will not be efficient. In fact, a previous study showed that the activation of the Nrf2 antioxidant response was not sufficient to counteract oxidative stress in affected MNs [47]. As a possible explanation, using the cell-type-specific expression profiles of Nrf2 it was shown that Nrf2 expression is enriched in astrocytes, compared to neurons [72], suggesting that the dysregulation of the Nrf2 pathway could affect the protective functions mediated by astrocytes. Interestingly, the chemical activation of Nrf2 in astrocytes was sufficient to provide antioxidant protection to MNs [63]. It follows that the development of tools to specifically target the Nrf2 pathway at the astrocyte level is highly required.

Another issue in drug development is related to the ALS animal models used, as therapeutics developed in mouse models almost always failed to translate in humans [119]. ALS animal models were established based on familial cases of ALS, using well-studied genes, such as SOD1, TDP-43, FUS, and C9ORF72. However, about 90% of ALS cases are sporadic and the underlying etiological events are still poorly characterized, so it is remains challenging to establish a validated animal model for sporadic ALS. As the absence of genetic targets in sporadic ALS patients limits the choice of treatments, the generation of animal models for sporadic ALS appears highly required to perform tests of drug candidates [100].

The drug development of Nrf2 activators against ALS is also limited by the need for a defined molecule to pass the blood–brain barrier, and not to show off-target toxicity or adverse effects due to its multifactorial mode of action. In this context, the validation of the interactions between chemical probes and their intended targets in vivo, as well as the correlation with drug efficacy and drug toxicity, appears as a critical step for the subsequent clinical translation [72].

For all these reasons, while Nrf2 modulating strategies represent a promising therapeutic approach to successfully treat ALS-affected patients, there is still a long way to go for novel Nrf2 activators to be used in patients. 

## 11. Conclusions

Studies around the world are ongoing to develop effective cures for ALS patients. There are different pathways impaired in ALS pathogenesis that could be the object of therapeutic strategies. In this frame, the Nrf2-mediated antioxidant response could be used as strategy to counteract chronic oxidative stress in ALS patients. Even though the road towards the discovery and clinical use of novel drugs still seems long, the FDA approval of edaravone, although its efficacy is very limited, supports the therapeutic potential of Nrf2 activators.

## Figures and Tables

**Table 1:** Oxidative stress markers in ALS animal models and patients.

Markers of Oxidative Stress	Experimental Model	Ref.
- MDA accumulation (lipid peroxidation)	SOD1^G93A^ mouse model	[17]
- Increase levels of MDA (lipid peroxidation) - Increased levels of 8-OHdG (oxidative DNA damage) - Increased GSSG/GSH ratio (oxidative stress).	Blood samples of SALS patients	[18]
- Increased levels of 4-HNE (lipid peroxidation)	Post-mortem specimens of lumbar spinal cord and/or occipital cortex	[19]
- Decreased GSH levels (oxidative stress)	Motor cortex of ALS patients	[20]
- Increased levels of urinary 8-OhdG and IsoP (lipid peroxidation)	Urine of SALS patients	[21]
- Increased HNE level (lipid peroxidation)	CSF of SALS patients	[22]
- Increased HNE level (lipid peroxidation)	Serum and CSF of SALS patients	[23]
- Increased protein carbonyl levels	Serum of SALS patients	[24]
- Increased levels of adducts of HNEH and CRAL (lipid peroxidation) - Increased levels of CML and pentosidine (protein glycoxidation)	Postmorten spinal cords of SALS patients	[25]
- Increase 8-OHG levels (DNA oxidation)	Postmortem brain and spinal cord tissues of ALS patients and SOD1^G93A^ mouse model	[26]

CML, N(epsilon)-(carboxymethyl)lysine; CRAL: crotonaldehyde-lysine; CSF: cerebrospinal fluid; HNE: 4-hydroxy-2-nonenal; HNEH, 4-hydroxy-2-nonenal-histidine; IsoP: 5-F2t-isoprostane; MDA: malondialdehyde; 8-OhdG: 8-hydroxy-2′-deoxyguanosine; 8-OHG: 8-hydroxyguanine.

**Table 2:** Preclinical and clinical studies of promising Nrf2-activators.

Mechanism of Nrf2 Activation	Compound	Model and trial	Outcome	Ref.
Electrophilic inhibition of Keap1-mediated Nrf2 degradation	Curcumin derivatives	SOD1^H46R^ mouse model	Improved motor function	[110]
		Clinical trial NCT04499963 (ongoing)		
		Clinical trial as add-on therapy to riluzole (completed)	Increased probability of survival without changes in motor function.	[43]
	Resveratrol	SOD1^G93A^ mouse model	Conflicting preclinical results	[111]
	Combined treatment of resveratrol and curcumin	Clinical trial NCT04654689 (ongoing)		
	CC100	Clinical trial NCT03049046 (completed)	Short-term treatment is safe and tolerable.	[101]
	DMF	Clinical trial as add-on to riluzole therapy ACTRN12618000534280 (completed phase II)	No improvement of survival and respiratory function	[81,98]
p62-mediated Keap1 degradation	Trehalose	SOD1^G86R^ mouse model	Increased survival and attenuated disease progress in mouse models.	[112]
		SOD1^G93A^ mouse model	Postponed disease onset, slowed down disease progress, no changes in survival.	[113]
		Clinical trial NCT05136885 (ongoing)		
	Rapamycin	p62 knockdown zebrafish model	Imrpoved motor function	[114]
		TDP-43 Drosophila model	Partially improved survival and motor function	[115]
		Clinical trial NCT03359538 (ongoing)		
Inhibition of GSK3β-promoted Nrf2 degradation by phosphorylation	Tideglusib	TDP-43 transgenic mice	Reduced TDP-43 phosphorylation in the spinal cord of TDP-43 transgenic mice.	[116]
		Clinical trial NCT05105958 (ongoing).		
	Lithium	Clinical studies (completed)	Pilot study NCT00818389 showed slowing ALS progression.	[108]
			Combined treatment of lithium and riluzole NCT00818389 did not show any effect	[117]
Unknown	Edaravone	FDA-approved drug in USA Canada and some other countries	Reduced level of oxidative stress and imrpoved motor and respiratory function.	[87]

## Data Availability

Not applicable.
